# Time-restricted feeding attenuates hypercholesterolaemia and atherosclerosis development during circadian disturbance in APOE∗3-Leiden.CETP mice

**DOI:** 10.1016/j.ebiom.2023.104680

**Published:** 2023-06-23

**Authors:** Wietse In Het Panhuis, Milena Schönke, Melanie Modder, Hannah E. Tom, Reshma A. Lalai, Amanda C.M. Pronk, Trea C.M. Streefland, Linda W.M. van Kerkhof, Martijn E.T. Dollé, Marie A.C. Depuydt, Ilze Bot, Winnie G. Vos, Laura A. Bosmans, Bram W. van Os, Esther Lutgens, Patrick C.N. Rensen, Sander Kooijman

**Affiliations:** a Division of Endocrinology, Department of Medicine, Leiden University Medical Center, Leiden, the Netherlands; b Einthoven Laboratory for Experimental Vascular Medicine, Leiden University Medical Center, Leiden, the Netherlands; c Centre for Health Protection, National Institute for Public Health and the Environment, Bilthoven, the Netherlands; d Leiden Academic Centre for Drug Research, Division of Biotherapeutics, Leiden University, Leiden, the Netherlands; e Department of Medical Biochemistry, Amsterdam UMC, Location AMC, University of Amsterdam, Amsterdam, the Netherlands; f Amsterdam Cardiovascular Sciences, Atherosclerosis & Ischemic Syndromes, Amsterdam, the Netherlands; g Amsterdam Immunity and Infection, Amsterdam, the Netherlands; h Department of Cardiovascular Medicine, Mayo Clinic, Rochester, MN, USA

**Keywords:** Circadian disturbance, Time-restricted feeding, Lipoprotein metabolism, Inflammation, Atherosclerotic cardiovascular disease

## Abstract

**Background:**

Circadian disturbance (CD) is the consequence of a mismatch between endogenous circadian rhythms, behaviour, and/or environmental cycles, and frequently occurs during shift work. Shift work has been associated with elevated risk for atherosclerotic cardiovascular disease (asCVD) in humans, but evidence for the effectiveness of prevention strategies is lacking.

**Methods:**

Here, we applied time-restricted feeding (TRF) as a strategy to counteract atherosclerosis development during CD in female APOE∗3-Leiden.CETP mice, a well-established model for humanized lipoprotein metabolism. Control groups were subjected to a fixed 12:12 h light–dark cycle, while CD groups were subjected to 6-h phase advancement every 3 days. Groups had either *ad libitum* (AL) access to food or were subjected to TRF with restricted food access to the dark phase.

**Findings:**

TRF did not prevent the increase in the relative abundance of circulating inflammatory monocytes and elevation of (postprandial) plasma triglycerides during CD. Nonetheless, TRF reduced atherosclerotic lesion size and prevented an elevation in macrophage content of atherosclerotic lesions during CD, while it increased the relative abundance of anti-inflammatory monocytes, prevented activation of T cells, and lowered plasma total cholesterol levels and markers of hepatic cholesterol synthesis. These effects were independent of total food intake.

**Interpretation:**

We propose that time restricted eating could be a promising strategy for the primary prevention of asCVD risk in shift workers, which warrants future study in humans.

**Funding:**

This work was funded by the 10.13039/501100009708Novo Nordisk Foundation, the Netherlands Ministry of Social Affairs and Employment, 10.13039/100019741Amsterdam Cardiovascular Sciences, and the 10.13039/100002129Dutch Heart Foundation.

**Research in context**

**Evidence before this study**

Circadian rhythms play a pivotal role in health. Therefore, it is not surprising that circadian disturbance (CD), a prominent feature of modern-day society with shift work as a prime example, increases atherosclerotic cardiovascular disease (asCVD) risk. Mistimed food intake is one of the proposed mechanisms contributing to elevated disease risk.

**Added value of this study**

Here, we subjected atherosclerosis-prone APOE∗3-Leiden.CETP mice to shifting light–dark cycles to induce CD while providing *ad libitum* food access or time-restricted feeding (TRF) with food access only during the dark phase. We show that TRF attenuates atherosclerosis development during CD, which coincided with increased relative abundance of circulating anti-inflammatory monocytes, a prevention of T cell activation, and lowered plasma total cholesterol levels and markers of hepatic cholesterol synthesis.

**Implications of all the available evidence**

We demonstrate that TRF attenuates atherosclerosis progression during CD, and provide insight in mechanisms underlying this protective effect. We propose that time-restricted eating could be a promising strategy for the primary prevention of asCVD risk in shift workers, which recently was demonstrated to be a feasible and safe dietary strategy for shift workers.

## Introduction

Cardiovascular diseases (CVDs) are the leading cause of mortality with approximately 30% of global deaths attributed to cardiovascular events.^1^ The main pathology underlying CVD is atherosclerosis, which is the result of progressive accumulation of lipids and immune cells and chronic low-grade inflammation in arteries. Current strategies to treat atherosclerotic (as)CVD focus on traditional risk factors, such as hypertension and dyslipidaemia. Despite their effectiveness, a considerable residual risk remains that may be attributed to non-traditional risk factors, including circadian disturbance (CD) that is caused by misalignment between circadian rhythms.^2^

Circadian rhythms are generated by a transcription-translation feedback loop, which is present in all cells of the body and under control of the suprachiasmatic nucleus (SCN). The SCN receives information about environmental light, and light thereby finetunes rhythms and allows for alignment of these rhythms with and adaptation to seasonal changes in environmental light–dark cycles. Light is accordingly referred to as the main *Zeitgeber*. This way, the circadian system regulates, for example, energy metabolism in a time of day-dependent manner in anticipation of daily variation in nutrient availability and requirements. However, modern society behaviour does often not align with physiological circadian rhythms, resulting in CD, which has been associated with increased risk for cardiometabolic diseases in humans.^3^, ^4^, ^5^, ^6^, ^7^, ^8^ We previously demonstrated that CD is causally linked to accelerated atherosclerosis development in APOE∗3-Leiden.CETP mice.^9^ The APOE∗3-Leiden.CETP mouse expresses a naturally occurring mutant form of human ApoE3 in addition to human ApoC1, which delays the hepatic clearance of lipoprotein remnants.^10^^,^^11^ In addition, the mouse is transgenic for human cholesteryl ester transfer protein (CETP), which transfers cholesteryl esters from high-density lipoprotein (HDL) to (very-) low-density lipoprotein (V)LDL particles and for which rodents are naturally deficient. The resulting effect is that the APOE∗3-Leiden.CETP mouse displays a human-like lipoprotein profile when fed a cholesterol-containing diet, and in contrast to other available mouse models for atherosclerosis responds well to lipid-lowering medication such as statins and proprotein convertase subtilisin/kexin type 9 (PCSK9) inhibitors.^11^^,^^12^ Given the increasing prevalence of severe CD as a result of large-scale shift work, effective forms of primary prevention to minimize the consequences of CD on asCVD risk are highly warranted.

A potential non-pharmacological approach to prevent cardiovascular problems associated with CD is time-restricted eating (TRE), which is an emerging paradigm that restricts food intake to a pre-defined shortened daily time-window and is referred to as time-restricted feeding (TRF) when applied in animal studies. In humans, TRE promotes weight loss and improves cardiometabolic health irrespective of ethnicity.^13^^,^^14^ In mice, TRF has additionally been shown to dictate diurnal gene expression patterns of peripheral tissues, such as the liver independently of light–dark cycles, demonstrating that food intake also acts as a *Zeitgeber*.^13^^,^^15^ In addition, we previously demonstrated that TRF can accelerate adaptation to changes in the light–dark cycle in mice.^16^ Based on these data, we hypothesized that TRF can aid in the protection from cardiometabolic disorders caused by CD. In the current study, we demonstrate that TRF attenuates the development of hypercholesterolaemia and atherosclerosis in APOE∗3-Leiden.CETP mice subjected to CD.

## Methods

### Animals

Female APOE∗3-Leiden.CETP mice were used, as in male mice plasma cholesterol levels do not sufficiently increase in response to a cholesterol-containing Western-type diet to promote atherogenesis. This sexual dimorphism relates to the inability of male mice to increase the rate of hepatic VLDL production in response to the diet, which is believed to be driven by female sex hormones.^17^ APOE∗3-Leiden.CETP mice were generated at the animal facility of the Leiden University Medical Center as previously described.^11^ Mice were housed in groups of 2–3 animals per cage in cabinets equipped with diffuse white fluorescent light (550–600 lux; Bailey True-Light fluorescent tubes, 139985, Technische Unie, Alphen aan de Rijn, the Netherlands) at 21 °C. Five weeks prior to the start of the intervention, the mice (7–15 weeks of age) were switched to Western-type diet containing 16% fat and 0.10% cholesterol (Diet T; Ssniff-Spezialdiäten GmbH, Soest, Germany), and mice continued to receive this diet throughout the intervention. After this adjustment period of 5 weeks, mice were randomized to one of four intervention groups (RandoMice software, Leiden, the Netherlands^18^). Control groups were subjected to a fixed 12:12 h light–dark cycle, while CD groups were subjected to a phase advancement of 6 h (i.e., shortening of the dark phase by 6 h) every 3 days. Shifting light–dark cycles is a well-used model to induce CD.^8^ Groups had either unrestricted (*ad libitum*, AL) access to food, or the access was restricted to the dark phase (TRF) using an automated feeding system (FeedTime, TSE-Systems GmbH, Berlin, Germany). The resulting four experimental groups (n = 16 mice per group) were therefore defined as Control + AL, CD + AL, Control + TRF, CD + TRF. Blinding of the group assignment was not possible due to the nature of the intervention. AL and TRF groups were housed in the same light cabinets. Control and CD groups were housed in different cabinets due to the differences in light–dark cycles.

All measurements were conducted on the day after a shift in light–dark cycle for the CD groups, and only when the light–dark cycles of the experimental groups were aligned (occurring every 11 days after four consecutive phase shifts in de CD group), unless indicated otherwise. Body weight was monitored using a scale and body composition was determined with EchoMRI (EchoMRI 100-Analyzer; EchoMRI, Houston, Texas). Blood was collected from the tail vein at indicated time points to measure plasma lipid levels. Additional blood was collected during days 23–24 to determine diurnal variation in plasma lipid levels relatively early in the study to select time points for follow-up measurements. A pre-defined subset of mice (n = 8 per group) was fasted for 4 h and subjected to a lipid tolerance test starting at ZT12 on day 67. Abundance of circulating immune cells was determined at two time points (n = 8 per group per time point) during days 94–95. After 100 days, mice were injected at two time points (n = 8 per group per time point; time difference between the first and last mouse was approximately 2 h for each time point) with radiolabelled TRL-mimicking particles. Mice were killed with CO_2_ 15 min after the injection, and subsequently perfused transcardially with ice-cold PBS for 5 min before organs were collected to assess plasma clearance and organ uptake of TRL-mimicking particles, and for other analyses. One mouse of the CD + AL group died prematurely after fighting, and three mice of the Control + TRF and two mice of the CD + TRF group were excluded from the analysis due to technical failure of the automated feeding systems.

### Voluntary physical activity and rhythm strength

Passive infrared sensors were used to measure activity of the mice within their home-cage during days 12–23 to assess rhythms in voluntary activity early during the intervention. Passive infrared sensor data were used to generate representative actograms to construct F-periodograms using Clocklab software version 6.1.05 (Actimetrics Software, Wilmette, Illinois, USA). Rhythm strength was derived from the amplitude of corresponding periodograms.^19^^,^^20^

### Indirect calorimetry

Energy expenditure was assessed in home-cages by means of indirect calorimetry (Promethion System; Sable Systems, Las Vegas, Nevada, USA) during days 61–63. Data on voluntary locomotor activity (by infrared beam breaks), food intake, O_2_ consumption, and CO_2_ production were continuously collected in 5-min bins, and energy expenditure was calculated. Sleep was estimated from voluntary locomotor activity and defined as inactivity >40 consecutive seconds. Data of two consecutive light–dark cycles was analysed, starting directly after the phase advancement in the CD group.

### Atherosclerosis quantification

Hearts were excised, fixated in 4% formaldehyde for 24 h and stored in 70% ethanol prior to embedding in paraffin and cross-sectioning using a microtome (5 μm sections). Cross sections were stained with haematoxylin-phloxine-saffron (HPS). Atherosclerotic lesion size of four cross sections throughout the aortic root with a distance of 50 μm, starting at open aortic valves, were quantified with imaging software (ImageJ version 1.52a; National Institutes of Health, Bethesda, Maryland), from which the mean atherosclerotic lesion area was calculated. In the same four cross sections, atherosclerotic lesions were scored subjectively for lesion type (I–V) and severity (Mild: type I-III lesions; Severe: type IV-V lesions), according to the guidelines of the American Heart Association adapted for mice, as previously described.^10^ Visual characteristics of each lesion type are listed in Table S1. Lesion type and severity were expressed as percentage of total lesions. This method is illustrated in Fig. S1. Monoclonal mouse antibody (1:1000; M0851; Dako, Heverlee, the Netherlands) against smooth muscle cell α-actin and secondary goat anti-mouse IgG (1:400; K4003; Dako, Heverlee, the Netherlands) were used to quantify smooth muscle cells. Quantification of macrophage content was performed by staining with rat monoclonal anti-mouse MAC-3 antibody (1:1000; 550292; BD Pharmingen, San Diego, USA) and secondary goat anti-rat IgG (MP7444; Vector Laboratories Inc., Burlingame, USA). The immunoperoxidase complexes on the secondary antibodies were visualized with Liquid Dab + Substrate Chromogen System (K3468, Dako, Heverlee, the Netherlands) and Nova Red (SK-4800, Vector Laboratories Inc., Burlingame, USA) for the smooth muscle cell and macrophage quantification, respectively. A solution of direct red (365548-5G) and fast green (F7258S) (both 1:1000; Sigma Aldrich, St. Louis, USA) was used to stain collagen. Smooth muscle cell-, collagen-, and macrophage-positive lesion area was quantified in all lesions from four cross sections (5 μm distance between different stainings) using a color threshold in ImageJ. Sample numbers were blinded prior to atherosclerosis quantification. Histological images representative of mean atherosclerotic lesion area, and total smooth muscle cell-, collagen-, and macrophage-positive area were selected for each group.

### Flow cytometry

Whole blood was incubated with hypotonic lysis buffer (160 mM ammonium chloride, 10 mM sodium bicarbonate, 1.3 mM ethylenediaminetetraacetic acid (EDTA); pH 7.4) to remove erythrocytes. Samples were resuspended and stained in staining buffer (0.5% bovine serum albumin, 5 mM EDTA in PBS; pH 7.4). Myeloid populations were identified using the following antibodies: αCCR2 (1:100, BV711, #747964, BD Biosciences, Franklin Lakes, New Jersey, USA), αCD11b (1:200, PerCP-Cy5.5, #550993, BD Biosciences), αCD11c (1:100, PE-Cy7, #25-0114, Thermo Fisher Scientific, Waltham, Massachusetts, USA), αCD16/32α (1:1000, #101330, BioLegend, San Diego, California, USA), αCD40 (1:100, PE, #124609, BioLegend), αCD45 (1:200, APC-Cy7, #103115, BioLegend), αCD86 (1:100, BV650, #105035, BioLegend), αLy6C (1:800, AF647, #128010, BioLegend), αLy6G (1:200, FITC, #11-5931, Thermo Fisher Scientific), αMHCII (1:200, BV510, #107635, BioLegend), and αSiglec-F (1:100, BV421, #562681, BD Biosciences). To identify T population, samples were stained with the following antibodies: αB220 (1:200, APC-eFluor780, #47-0452, Thermo Fisher Scientific), αCCR2 (1:100, BV711, #747964, BD Biosciences), αCD3 (1:100, BV510, #100353, BioLegend), αCD4 (1:800, BV650, 100469, BioLegend), αCD8 (1:1000, BV605, #100744, BioLegend), αCD16/32α (1:1000, #101330, BioLegend), CD19 (1:200, PerCP-Cy5.5, #45-0193, Thermo Fisher Scientific), αCD40 (1:100, PE, #124609, BioLegend), αCD44 (1:300, FITC, #103006, BioLegend), αCD62L (1:1000, PE-Cy7, #104418, BioLegend), and αCXCR3 (1:200, APC, #126511, BioLegend). Prior to analysis DAPI was added (final concentration of 15 ng/mL, D21490, Thermo Fisher Scientific) to exclude dead cells. Cells were measured on a LSRFortessa Cell Analyzer (BD Biosciences) and data were analysed using FCS Express software, version 7 (De Novo Software).

### Plasma measurements

Plasma TG and total cholesterol (TC) levels were measured utilizing enzymatic Cobas Triglycerides (106571) or Cobas Total Cholesterol (106570) kits (both from Roche Diagnostics, Mannheim, Germany), by combining 7.5 μL sample (5× diluted) with 200 μL reagent (reagent was undiluted for TG and 3 × diluted for TC) prior to incubation at room temperature for 30 min and measuring transmittance at 492 nm vs. 650 nm (for TG) or at 505 nm vs. 650 nm (for TC). To calculate total TC exposure, the area under the curve was determined from regularly monitored plasma TC levels over time.

### Postprandial lipid tolerance

Mice received an oral olive oil bolus via gavage (8 μL/g body weight per mouse) (Carbonell, Cordoba, Spain). Tail vein blood was sampled at indicated time points to measure plasma TG levels.

### Clearance of radiolabelled lipoprotein-like emulsion particles

Mice were intravenously injected with an emulsion of TG-rich lipoprotein-like particles (80 nm) that were double-labelled with glycerol tri [^3^H]oleate and [^14^C]cholesteryl oleate (1 mg TG in 200 μL saline per mouse), prepared as described previously.^21^ Blood was collected at indicated time points to determine plasma decay of radiolabels. Tissues (approx. 50–200 mg) were dissolved in 0.5 mL Solvable (6NE9100, PerkinElmer, Waltham, Massachusetts, USA) at 56 °C overnight, after which 5.0 mL Ultima Gold (6013329, PerkinElmer, Waltham, Massachusetts, USA) was added. Plasma (4 μL) was directly added to 2.5 mL Ultima Gold. ^3^H- and ^14^C-activity was measured with a liquid scintillation counter (Tri-Carb 2910 TR, PerkinElmer, Waltham, Massachusetts, USA) and expressed as a percentage of injected dose per whole tissue or as a percentage of injected dose in plasma. One mouse was excluded for calculating the AUC (between 2 and 15 min after injection) of plasma decay since one plasma sample was missing. This criterium was set *a priori* since a sample from each time point is required for the AUC calculation.

### Gene expression

RNA was isolated from frozen liver (approx. 30 mg) or aorta (aortas were collected starting at the ascending aorta until the diaphragm) by lysis and homogenization using TriPure RNA Isolation Reagent (11667165001, Sigma–Aldrich, Saint Louis, USA) and a FastPrep-24™ 5G bead beating grinder and lysis system (4.0 m/s for 10 s; MP Biomedicals™, Santa Ana, California, USA). cDNA was synthesized from 1 μg RNA using M-MLV Reverse Transcriptase (M1705, Promega, Madison, USA) and qPCR was conducted utilizing SYBR green kit (Promega, Madison, Wisconsin, USA) and a CFX96 PCR machine (Bio-Rad, Hercules, USA), according to the manufacturers’ protocols. Gene expression was normalized to glyceraldehyde-3-phosphate dehydrogenase (*Gapdh*) for liver and to β2 microglobulin (*B2m*) for aorta and expressed relative to the Control + AL group at ZT0. Primer sequences are provided in Table 1.

**Table 1:** Primer sequences.

Gene	Forward primer	Reverse primer
*Abcg5*	TGTCCTACAGCGTCAGCAACC	GGCCACTCTCGATGTACAAGG
*Adgre1*	CTTTGGCTATGGGCTTCCAGTC	GCAAGGAGGACAGAGTTTATCGTG
*Apob*	GCCCATTGTGGACAAGTTGATC	CCAGGACTTGGAGGTCTTGGA
*B2m*	TGACCGGCTTGTATGCTATC	CAGTGTGAGCCAGGATATAG
*Bmal1*	ATGCCAAGACTGGACTTCCG	TGCAGAAGCTTTTTCGATCTGC
*Ccr2*	TGCCATCATAAAGGAGCCA	AGCACATGTGGTGAATCCAA
*Clock*	AGTTAGGGCTGAAAGACGGC	GGTGTGGAGGAAGGGTCTGA
*Cyp7a1*	CAGGGAGATGCTCTGTGTTCA	AGGCATACATCCCTTCCGTGA
*Gapdh*	GGGGCTGGCATTGCTCTCAA	TTGCTCAGTGTCCTTGCTGGGG
*Hif1*	ACGAGAAGAAAAATAGGATGAGTTC	GTGGCAACTGATGAGCAAGC
*Hmgcr*	CCGGCAACAACAAGATCTGTG	ATGTACAGGATGGCGATGCA
*Icam1*	TCCGCTGTGCTTTGAGAACT	TCCGGAAACGAATACACGGT
*iNos*	CGGGCATCTGGTAGCCAGCG	TGGCAACATCAGGTCGGCCAT
*Mttp*	CTCTTGGCAGTGCTTTTTCTCT	GAGCTTGTATAGCCGCTCATT
*Per2*	TGTGTGCTTACACGGGTGTCCTA	ACGTTTGGTTTGCGCATGAA
*Sod1*	TACACAAGGCTGTACCAGTGC	ACATGCCTCTCTTCA TCCGC
*Srebp2*	TGAAGCTGGCCAATCAGAAAA	ACATCACTGTCCACCAGACTGC
*Tnf*	GCCTCTTCTCATTCCTGCTTG	CTGATGAGAGGGAGGCCATT
*Vcam1*	TGGAGGTCTACTCATTCCCTGA	GACAGGTCTCCCATGCACAA

### Ethics statement

Experiments were performed in accordance with the Institute for Laboratory Animal Research Guide for the Care and Use of Laboratory Animals and were approved by the National Committee for Animal Experiments of the Netherlands (AVD11600202010187) and by the Ethics Committee on Animal Care and Experimentation of the Leiden University Medical Center (PE.21.002.010). All animal procedures were conformed the guidelines from Directive 2010/63/EU of the European Parliament on the protection of animal used for scientific purposes.

### Statistical analyses

The primary outcome was atherosclerotic lesion size, of which we anticipated a 40% difference to be biologically relevant and expected a standard deviation of 33%. Therefore, 16 mice per group were needed with a power of 80%, an acceptable type I error rate of 5%, and four pair-wise comparisons. Significant outliers were identified with a Grubbs outlier test and removed from the analysis (α = 5%). Statistical analyses between groups were performed with unpaired t-tests, two-way or three-way analysis of variance (ANOVA) and following Tukey's multiple-comparison post-hoc test, where applicable, to describe main and interaction effects, as well as group-comparisons. Pearson product–moment correlation coefficients were determined to assess linear correlations between variables. For diurnal plasma TG and TC levels, cosinor analysis was performed using a fitting function with a 24-h period to obtain the acrophase and amplitude.^22^ P < 0.05 was considered statistically significant. Statistical analyses were performed with GraphPad Prism software, version 9.3.1 (GraphPad, La Jolla, California). Data are presented as individual data points and/or as means ± SD.

### Role of funders

Funders did not participate in the study design, data collection, data analyses, interpretation, or writing of the manuscript.

## Results

### TRF does not change behaviour or whole-body energy balance during CD

**Fig. 1:**
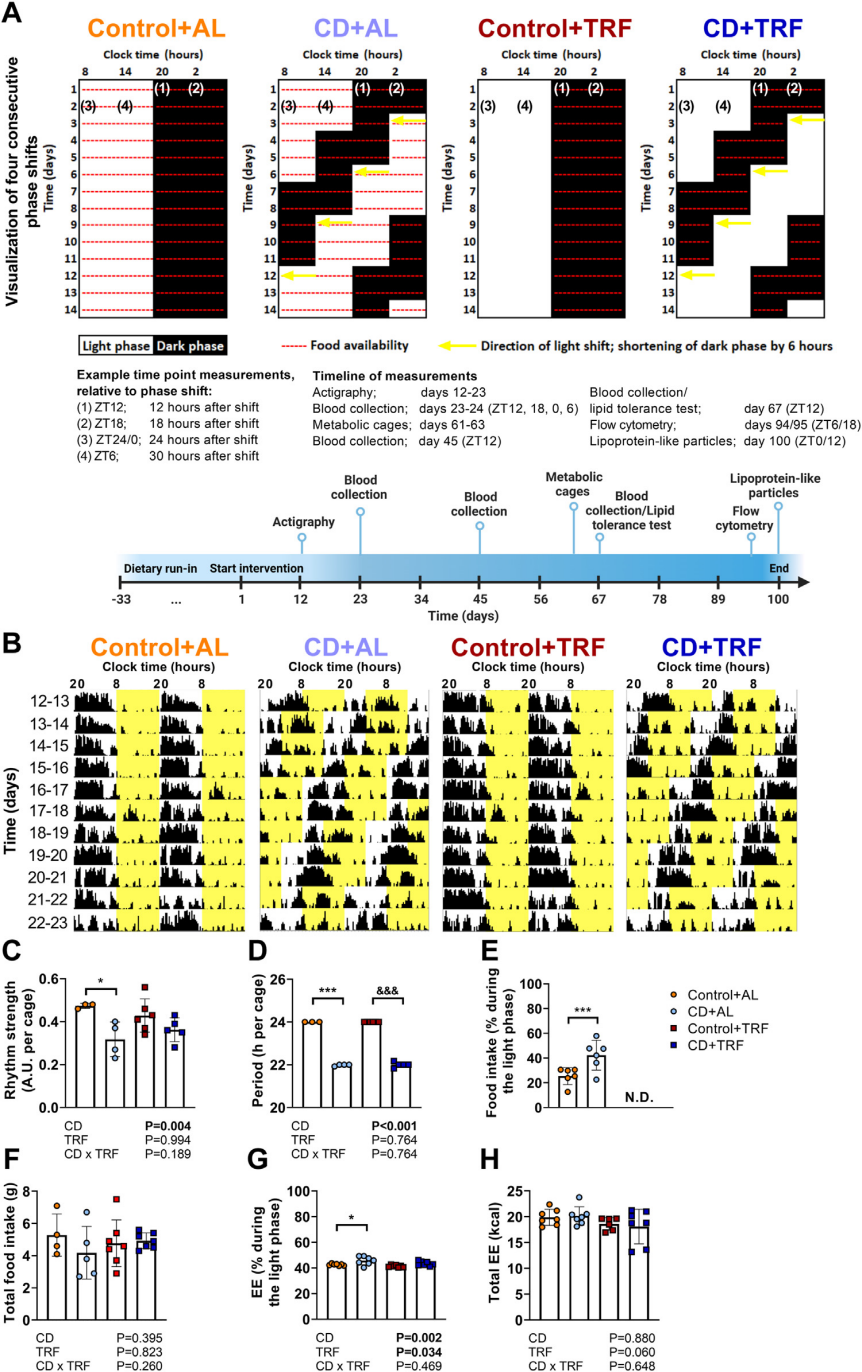
**Study setup, rhythms of behaviour, and energy balance.** APOE∗3-Leiden.CETP mice were exposed to 6-h phase advancement every 3 days (circadian disturbance; CD) or regular 12:12 light–dark cycle (Control), while having either *ad libitum* food access (AL) or food access during the dark phase only (time-restricted feeding; TRF). (**A**) The shifts in light–dark cycle are displayed for the first 11 days, covering four shifts. Mice were subjected to the intervention for a total duration of 14 weeks. Prior to the first shift, mice were subjected to a dietary run-in period for 22 days on a normal light–dark cycle. A timeline is provided displaying *in vivo* measurements with corresponding duration or time point(s) (in *Zeitgeber* Time (ZT)). (**B**) Actograms of four consecutive shifts during days 12–23 of the study were constructed (and double-plotted) and periodogram analysis was used to calculate (**C**) rhythm strength (expressed as A.U., arbitrary unit) and (**D**) period (n = 3–6 cages of 2–3 mice/group). During days 61–63, mice were housed in metabolic home-cages for continuous measurement of (**E, F**) food intake and (**G, H**) energy expenditure (EE), after which the percentage of each parameter during the light phase and the total levels were calculated (n = 4–7 cages of 2–3 mice/group). Data are presented as means ± SD. ∗ Control + AL vs. CD + AL; ^&^Control + TRF vs. CD + TRF. ∗p < 0.05; ∗∗∗^,&&&^ p < 0.001, according to unpaired t-test (D) or two-way ANOVA and following Tukey's multiple-comparison test (B–C, E–G). (A) was created with BioRender.com.

**Fig. 2:**
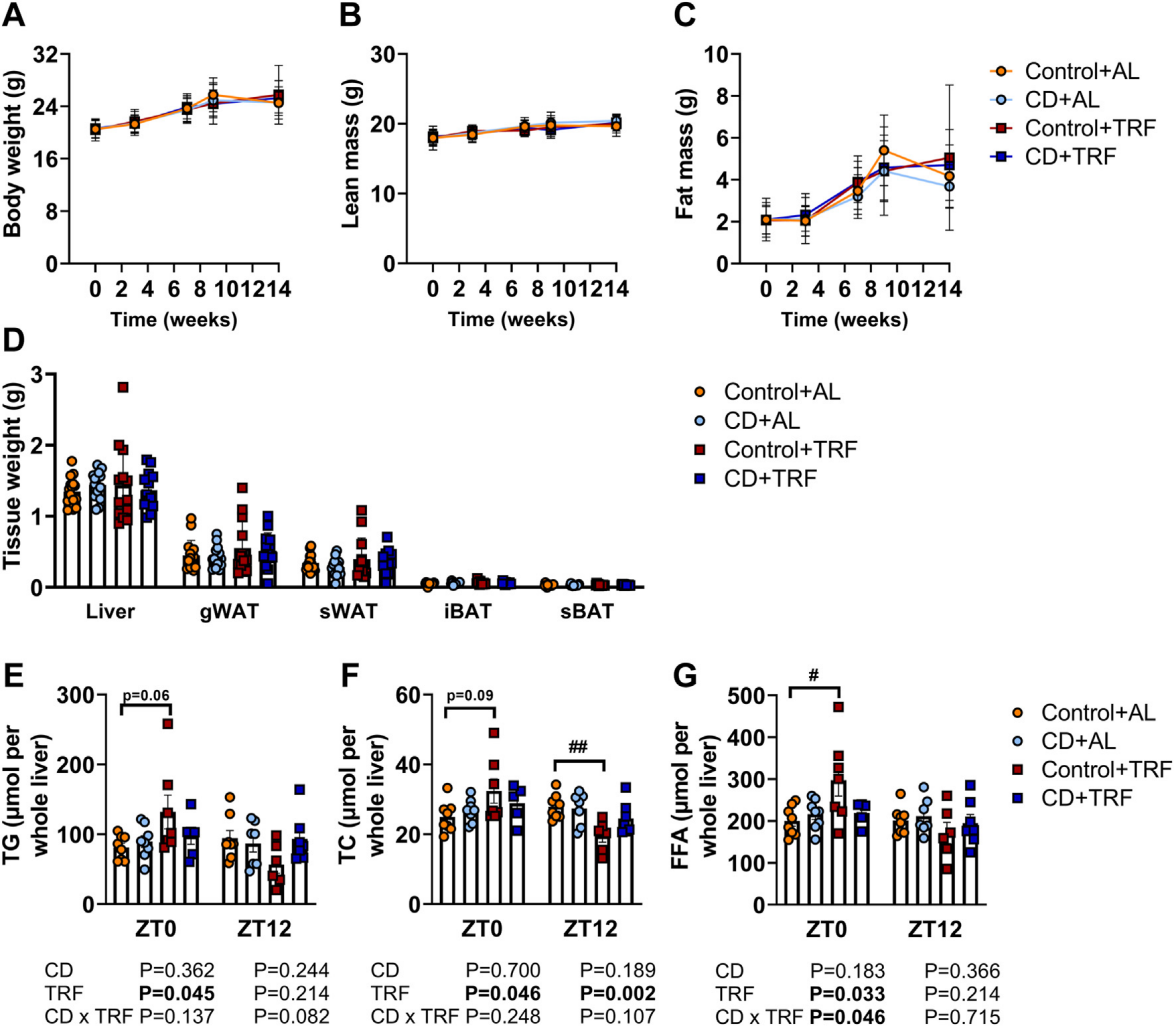
**Body composition, tissue weights, and hepatic lipid content.** APOE∗3-Leiden.CETP mice were exposed to 6-h phase advancement every 3 days (circadian disturbance; CD) or regular 12:12 light–dark cycle (Control), while having either *ad libitum* food access (AL) or food access during the dark phase only (time-restricted feeding; TRF) for a total duration of 14 weeks. (**A**) Body weight, (**B**) lean mass, and (**C**) fat mass were monitored throughout the study. (**D**) Weight of liver, gonadal white adipose tissue (gWAT), subcutaneous WAT (sWAT), interscapular brown adipose tissue (iBAT) and subscapular BAT (sBAT) (n = 13–16 mice/group) was assessed at the end of the study. Lipids were extracted from livers collected at *Zeitgeber* time (ZT) 0 and 12 to measure (**E**) triglycerides (TG), (**F**) total cholesterol (TC), and (**G**) free fatty acids (FFA). Data are presented as means ± SD. ^#^Control + AL vs. Control + TRF. ^#^p < 0.05; ^##^P < 0.01, according to two-way ANOVA and following Tukey's multiple-comparison test.

APOE∗3-Leiden.CETP mice were subjected to a fixed 12:12 h light–dark cycle (Control groups) or to 6-h light phase advancement every 3 days (CD groups). The study setup is displayed in Fig. 1a. CD was confirmed through monitoring of voluntary physical activity during days 12–23 (covering four consecutive phase shifts) (Fig. 1b), and from which rhythm strength (Fig. 1c) and period (Fig. 1d) were calculated. TRF seemed to partly prevent the reduction in rhythm strength caused by CD, although rhythm strength in the CD + TRF group was not statistically different from CD + AL. We next assessed diurnal food intake and whole-body energy balance during days 61–63. Two consecutive 12:12 h light–dark cycles were analysed, starting directly after the phase advancement in the CD groups. CD increased relative voluntary locomotor activity during the light phase while increasing total activity, regardless of TRF, and without altering estimated sleep (Fig. S2a–d). CD caused a shift in food intake and energy expenditure from the dark phase to the light phase (i.e., a relative increase during the light phase), without altering total daily levels (Fig. 1e–h). In line with rhythm strength, TRF partly prevented the shift in energy expenditure from the dark to the light phase seen with CD, but without significant differences between the CD + TRF and CD + AL group (Fig. 1g). CD did not alter body weight and composition (Fig. 2a–c), tissue weights of liver, white adipose tissue (WAT), and brown adipose tissue (BAT) (Fig. 2d), or hepatic lipid content (Fig. 2e–g), regardless of TRF. Interestingly, TRF induced variation between ZT0 and ZT12 in hepatic lipid content when mice were exposed to normal light–dark cycles but not during CD (Fig. 2e–g).

### TRF attenuates atherosclerosis lesion size and macrophage content during CD

**Fig. 3:**
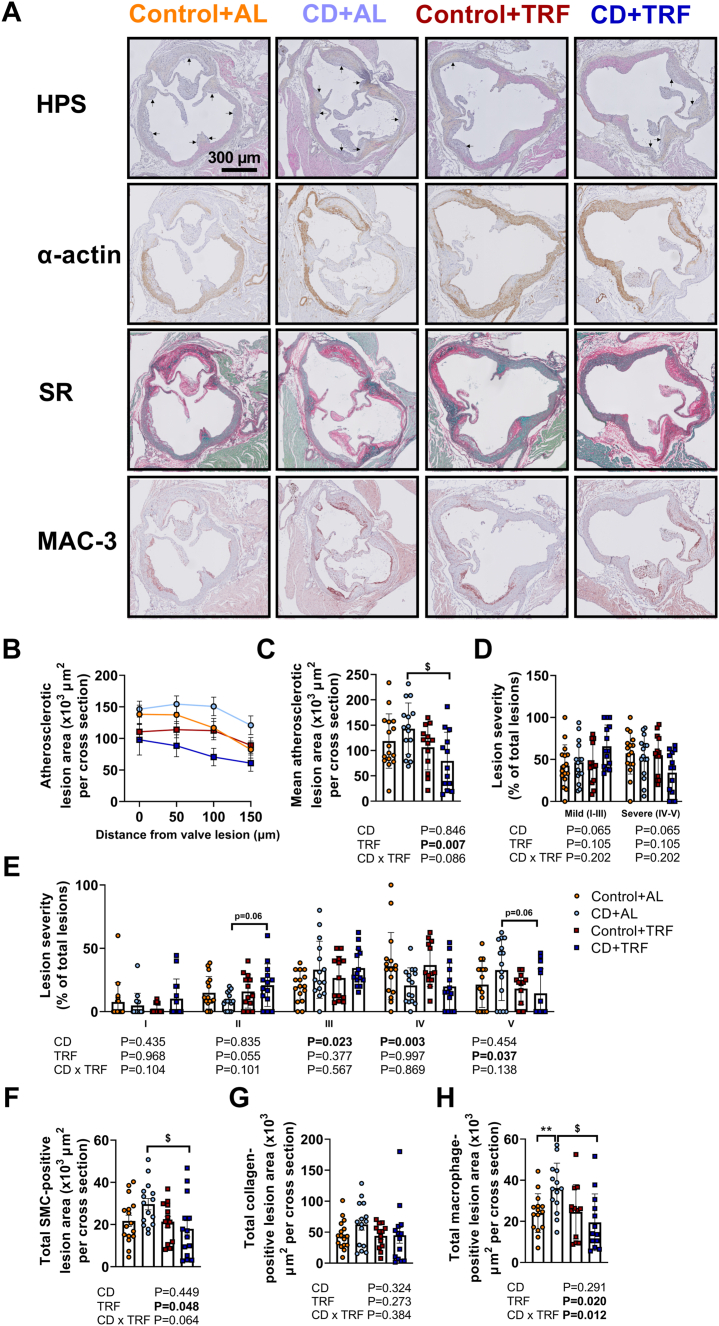
**Atherosclerotic lesion size, severity, and composition.** APOE∗3-Leiden.CETP mice were exposed to 6-h phase advancement every 3 days (circadian disturbance; CD) or regular 12:12 light–dark cycle (Control), while having either *ad libitum* food access (AL) or food access during the dark phase only (time-restricted feeding; TRF) for a total duration of 14 weeks after which hearts were collected. Cross sections of the aortic root area were stained with (**A**) haematoxylin-phloxine-saffron (asterisks are positioned in lumen next to lesions) or with anti-α-actin antibody, Sirius Red (SR), or anti-MAC-3 antibody, and atherosclerotic lesion area was determined and (**B**) expressed as a function of distance from the appearance of open aortic valves, from which (**C**) the mean atherosclerotic lesion area was calculated. Lesions were categorized according to lesion severity, expressed as a percentage of total lesions, and shown (**D**) per lesion severity (mild; type I-III and severe; type IV-V) and (**E)** lesion type (I–V). Using anti-α-actin antibody, SR, and anti-MAC-3 antibody-stained cross sections, the (**F**) smooth muscle cell (SMC)-positive lesion area, (**G**) collagen-positive lesion area, and the (**H**) macrophage-positive lesion area was calculated (n = 12–16 mice/group). Data are presented as means ± SD. ^$^CD + AL vs. CD + TRF; ∗ Control + AL vs. CD + AL. ^$^ P < 0.05; ∗∗P < 0.01, according to two-way ANOVA and following Tukey's multiple-comparison test.

Next, we evaluated atherosclerosis development in the aortic root, by assessing lesion area, severity, and composition including macrophage, smooth muscle cell, and collagen content. Macrophages play a role in atherosclerosis formation by engulfing cholesterol-rich lipoproteins trapped in the intima, causing subsequent formation of cholesterol-containing foam cells.^23^ In advanced lesions referred to as atheroma, smooth muscle cells differentiate from the tunica media to the intima and proliferate to form a fibrotic cap on the luminal side, together with collagen that they synthesize. While CD did not significantly increase atherosclerotic lesion area (Fig. 3a–c) or alter lesion severity, smooth muscle cell- or collagen-positive lesion area (Fig. 3a and d–g), the CD + AL group showed significantly increased macrophage-positive lesion area compared with Control + AL (Fig. 3a and h). In mice subjected to CD, TRF reduced atherosclerotic lesion area (Fig. 3a–c) and without notably altering lesion severity (Fig. 3d and e), while lowering smooth muscle cell-positive lesion area, but not collagen-positive lesion area (Fig. 3a and f–g). Strikingly, TRF fully prevented the elevation in macrophage content in lesions caused by CD (Fig. 3a and h). These findings suggest that TRF attenuates lesion development during CD.

### TRF increases anti-inflammatory monocytes and attenuates the activated T cell profile caused by CD

**Fig. 4:**
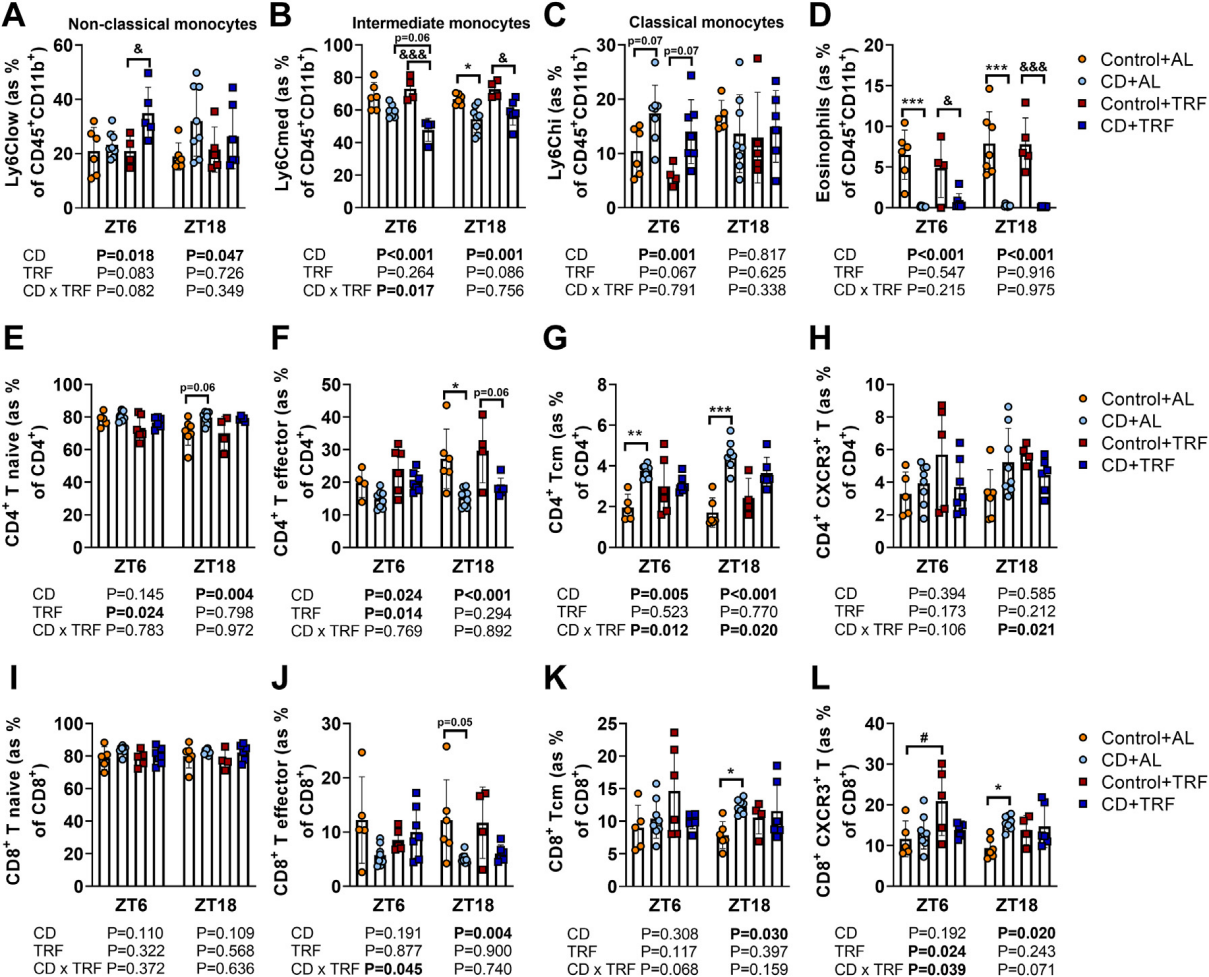
**Circulating monocytes, eosinophils, and T cells.** APOE∗3-Leiden.CETP mice were exposed to 6-h phase advancement every 3 days (circadian disturbance; CD) or regular 12:12 light–dark cycle (Control), while having either *ad libitum* food access (AL) or food access during the dark phase only (time-restricted feeding; TRF) for a total duration of 14 weeks. During days 94–95, blood was collected to measure abundance of circulating (**A**) non-classical monocytes (Ly6Clow), (**B**) intermediate monocytes (Ly6Cmed), (**C**) classical monocytes (Ly6Chigh), (**D**) eosinophils, (**E**) cluster of differentiation (CD)4^+^ T naive cells, (**F**) CD4^+^ T effector cells, (**G**) CD4^+^ T central memory (Tcm) cells, (**H**) CD4^+^ C-X-C Motif Chemokine Receptor 3 (CXCR3^+^) T cells, (**I**) CD8^+^ T naive cells, (**J**) CD8^+^ T effector cells, (**K**) CD8^+^ Tcm cells, and (**L**) CD8^+^ CXCR3^+^ T cells using flow cytometry (n = 4–8 mice/group/time point). Data are presented as means ± SD. ^#^Control + AL vs. Control + TRF; ∗ Control + AL vs. CD + AL; ^&^Control + TRF vs. CD + TRF. ^#,^∗^,&^ P < 0.05; ∗∗P < 0.01; ∗∗∗^,&&&^ P < 0.001, according to two-way ANOVA and following Tukey's multiple-comparison test.

To obtain further insight in the mechanisms underlying the atherogenic effect of CD, and via which mechanisms TRF attenuates this risk, we first assessed markers of local inflammation in the aortic vessel wall. To this end, we measured the expression of genes involved in inflammation (tumor necrosis factor alpha (*Tnf*), adhesion G protein-coupled receptor E1 (*Adgre1*), and inducible nitric oxide synthase (*iNos*)), leukocyte recruitment (intercellular adhesion molecule 1 (*Icam1*), vascular cell adhesion molecule 1 (*Vcam1*), and C-C chemokine receptor type 2 (*Ccr2*)), and oxidative stress (superoxide dismutase 1 (*Sod1*), and hypoxia inducible factor 1 subunit alpha (*Hif1*)) in the aorta (Fig. S3). However, for none of these genes differential expression was observed, suggesting no changes in local inflammation. We therefore continued with assessing markers of systemic inflammation, by measuring relative abundance of circulating immune cell populations, including monocytes, eosinophils, and T cells, as all of these cell types are involved in atherogenesis as explained below. The relative abundance of these cells were measured during days 94–95 at ZT6 and ZT18 (6 and 18 h after the last phase shift), as these time points correspond to the peak and trough levels of many immune cell populations in the blood of mice, respectively.^9^ We started with assessment of monocytes (CD45^+^ CD11b^+^ Ly6C^+^), which play an essential role in atherosclerosis development, and can be distinguished as non-classical (Ly6Clow), intermediate (Ly6Cmed), and classical (Ly6Chi) monocytes. Non-classical monocytes are considered to be anti-inflammatory, whereas classical monocytes are considered to be pro-inflammatory and can adhere to activated endothelium and infiltrate the vessel wall after which they differentiate into macrophages.^24^ Intermediate monocytes are a transition population, with characteristics of both classical and non-classical monocytes, but their role in atherosclerosis development in mice is poorly understood.^25^^,^^26^ Regardless of the feeding intervention, CD caused shifts in monocyte subtypes, by elevating the relative number of anti-inflammatory non-classical monocytes (CD effect at ZT18 by two-way ANOVA: P = 0.047) (Fig. 4a), lowering intermediate monocytes (CD effect by two-way ANOVA: P < 0.001 and P < 0.001 for ZT6 and ZT18, respectively) (Fig. 4b), and elevating pro-inflammatory classical monocytes (CD effect at ZT6 by two-way ANOVA: P = 0.001) (Fig. 4c). Compared with AL feeding, TRF tended to further elevate anti-inflammatory non-classical monocytes at ZT6 (CD-TRF interaction at ZT6 by two-way ANOVA: P = 0.082) (Fig. 4a) and further reduced intermediate monocytes at the same time point (CD-TRF interaction at ZT6 by two-way ANOVA: P = 0.017) (Fig. 4b), without preventing the elevation of classical monocytes caused by CD (Fig. 4c). These findings suggest that CD shifts the monocyte profile towards both anti-inflammatory non-classical and pro-inflammatory classical monocytes while reducing intermediate monocytes, and that TRF might be able to shift this balance towards more anti-inflammatory non-classical monocytes and less towards intermediate monocytes. Interestingly, eosinophils, which upon binding to the endothelium can promote leukocyte migration into the vessel wall,^27^ were depleted by CD regardless of TRF (CD effect by two-way ANOVA: P < 0.001 and P < 0.001 at ZT6 and ZT18, respectively) (Fig. 4d), suggesting either a systemic depletion of eosinophils or migration into tissue, among which atherosclerotic lesions.

Next, we looked at relative abundance of CD4^+^ T helper cells, of which the effector cells can be pro- or anti-atherogenic, depending on their polarization.^28^ Mice subjected to CD had a higher relative number of CD4^+^ T naive cells (CD44^−^ CD62L^+^) (i.e., mature cells that have not been activated) at ZT18 (CD effect at ZT18 by two-way ANOVA: P = 0.004) and a reduced number of effector CD4^+^ T cells (CD62L^−^) at ZT6 and ZT18 regardless of TRF (CD effect by two-way ANOVA: P = 0.024 and P < 0.001 for ZT6 and ZT18, respectively) (Fig. 4e and f). Interestingly, under AL feeding, CD resulted in a relative increase of CD4^+^ T central memory (Tcm) cells (CD44^+^ CD62L^+^) (i.e., cells without effector function that were formed upon activation of naive cells for augmented immune response upon reactivation) at ZT6 and ZT18 (Fig. 4g), which may indicate that T cells have been activated earlier in the process of atherosclerosis development. Notably, the increase in Tcm cells did not occur when mice were subjected to TRF during CD (CD-TRF interaction by two-way ANOVA: P = 0.012 and P = 0.020 for ZT6 and ZT18, respectively). Despite the reduction in effector CD4^+^ T cells, the subset of CD4^+^ T cells expressing C-X-C Motif Chemokine Receptor 3 (CXCR3), a chemokine receptor closely associated with the pro-atherogenic T helper 1 subset,^28^ was unchanged by CD (Fig. 4h). In contrast to CD4^+^ T cells, the role of CD8^+^ (cytotoxic) T cells in atherosclerosis is less well-understood.^28^ CD did not alter the relative number of CD8^+^ T naive cells, but did reduce CD8^+^ T effector cells at ZT18 (CD effect at ZT18 by two-way ANOVA: P = 0.004) whilst increasing CD8^+^ Tcm cells (CD effect at ZT18 by two-way ANOVA: P = 0.030) (Fig. 4i–k), again suggesting prior T cell activation. In line with CD4^+^ Tcm cells (Fig. 4g), TRF prevented the increase in CD8^+^ Tcm cells caused by CD (Fig. 4k). These findings might indicate that during the light–dark cycle shifting-intervention, CD caused an activated T cell profile in the blood with AL feeding but not with TRF. Interestingly, CD and TRF alone (CD and TRF effect by two-way ANOVA: P = 0.020 and P = 0.024 for CD at ZT18 and TRF at ZT6, respectively), but not in combination (CD-TRF interaction by two-way ANOVA: P = 0.039 and P = 0.071 at ZT6 and ZT18, respectively), increased the relative abundance of CXCR3^+^ cytotoxic T cells (Fig. 4l), which are considered to be pro-atherogenic.^28^ Collectively, these findings indicate that CD does not influence local aortic tissue inflammation, but rather induces systemic changes by shifting circulating monocyte subset, depletion of circulating eosinophils, and causing an activated T cell profile in blood. TRF partly prevented these effects by increasing anti-inflammatory monocytes and attenuating the activated T cell profile.

### TRF reduces plasma total cholesterol levels during CD

**Fig. 5:**
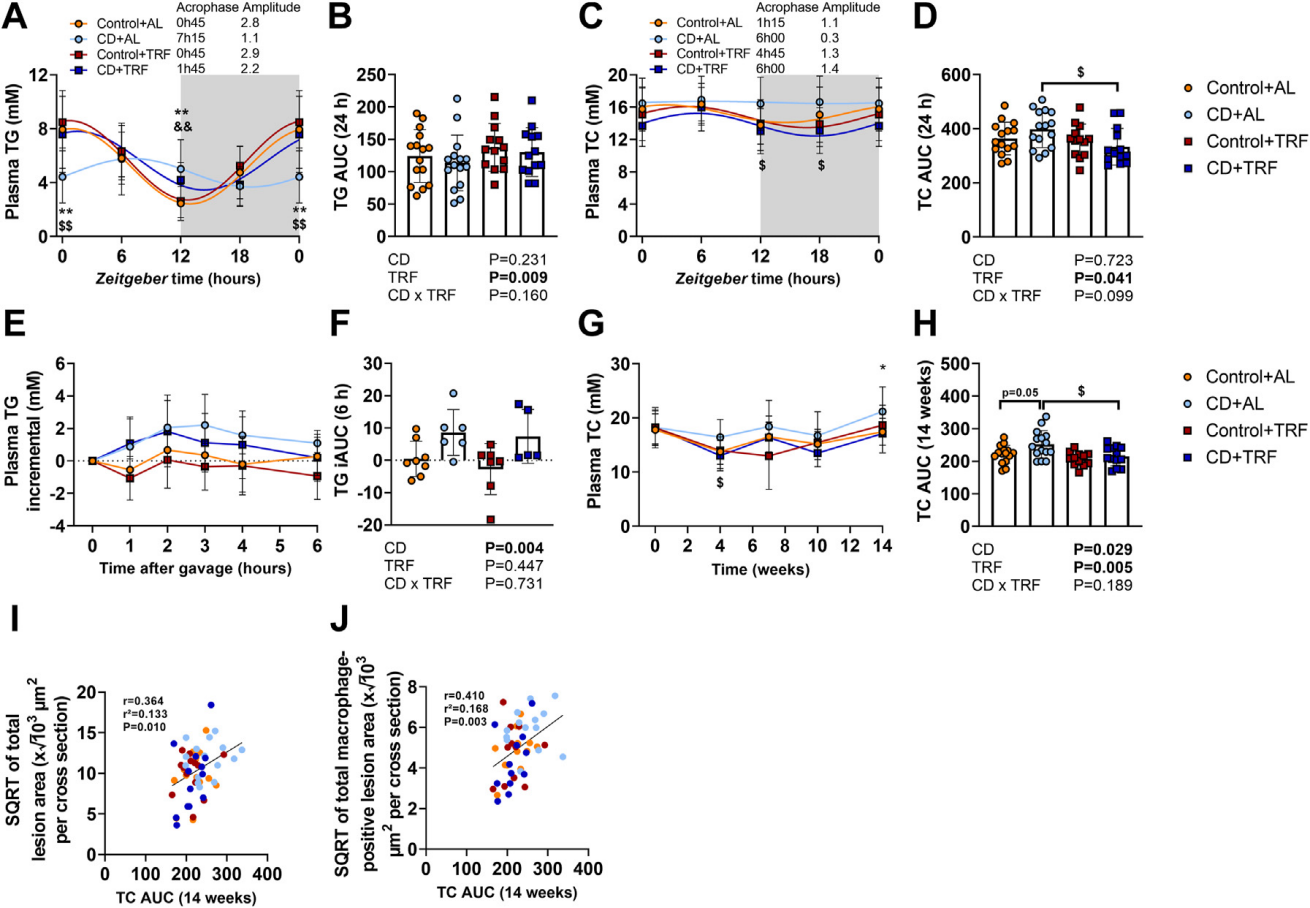
**Diurnal and postprandial plasma lipid levels.** APOE∗3-Leiden.CETP mice were exposed to 6-h phase advancement every 3 days (circadian disturbance; CD) or regular 12:12 light–dark cycle (Control), while having either *ad libitum* food access (AL) or food access during the dark phase only (time-restricted feeding; TRF) for a total duration of 14 weeks. During days 23–24, plasma was collected at *Zeitgeber* time (ZT) 0, 6, 12, and 18 to measure (**A**) triglycerides (TG) (**B**) from which the area under the curve (AUC) was calculated, and (**C**) total cholesterol (TC) (**D**) from which the AUC was calculated (n = 13–15 mice/group). ZT0 was double-plotted, and cosinor analysis was used to plot sinewaves and calculate acrophase and amplitude. On day 67, mice received an oral olive oil bolus to measure postprandial (**E**) plasma TG levels (**F**) from which the AUC was calculated (n = 5–8 mice/group). (**G**) Plasma TC levels were monitored throughout the study (**H**) from which the AUC was calculated (n = 12–15 mice/group). The total cholesterol exposure throughout the study (TC AUC) was plotted against the square root of the (**I**) total lesion area and (**J**) total MAC-3 positive lesion area from which B and Pearson correlation coefficients were determined. Data are presented as means ± SD. ^$^CD + AL vs. CD + TRF; ∗ Control + AL vs. CD + AL; ^&^Control + TRF vs. CD + TRF. ^$^ P < 0.05; ^&&,^∗∗P < 0.01, according to two-way ANOVA and following Tukey's multiple-comparison test.

We next focused on plasma lipids that are tightly linked to atherosclerosis development,^29^ and show large diurnal variation. Plasma lipid levels were assessed during days 23–24 at ZT0, ZT6, ZT12, and ZT18 (24, 30, 12, and 18 h after the last phase shift for ZT0, ZT6, ZT12, and ZT18, respectively). CD attenuated diurnal rhythmicity of plasma TG and TC levels (time effect by one-way ANOVA: P = 0.103 and P = 0.992 for TG and TC, respectively), while shifting the acrophase and lowering amplitude (Fig. 5a and c, Tables S2 and S3). While TRF by itself slightly elevated total plasma TG levels (TRF effect by two-way ANOVA: P = 0.009) (Fig. 5b), TRF prevented the attenuations in diurnal plasma TG and TC levels caused by CD (time effect by one-way ANOVA: P < 0.001 and P = 0.040 for TG and TC, respectively) (Fig. 5a and c, Tables S2 and S3) and lowered total plasma TC levels during CD (Fig. 5c and d). We next performed an oral lipid tolerance test at the time point when TG levels were elevated in the CD groups (i.e., ZT12, 12 h after the last phase shift) to find CD delayed postprandial TG excursions, regardless of TRF (CD effect by two-way ANOVA: P = 0.004) (Fig. 5e and f). Notably, CD increased TC exposure throughout the study (CD effect by two-way ANOVA: P = 0.029) and TRF reduced TC exposure in mice subjected to CD (Fig. 5g and h). Importantly, TC exposure throughout the study correlated with atherosclerotic lesion area and with the macrophage-positive lesion area (Fig. 5i and j). These data suggest that TRF counteracts atherosclerosis development by attenuating hypercholesterolaemia during CD.

**Fig. 6:**
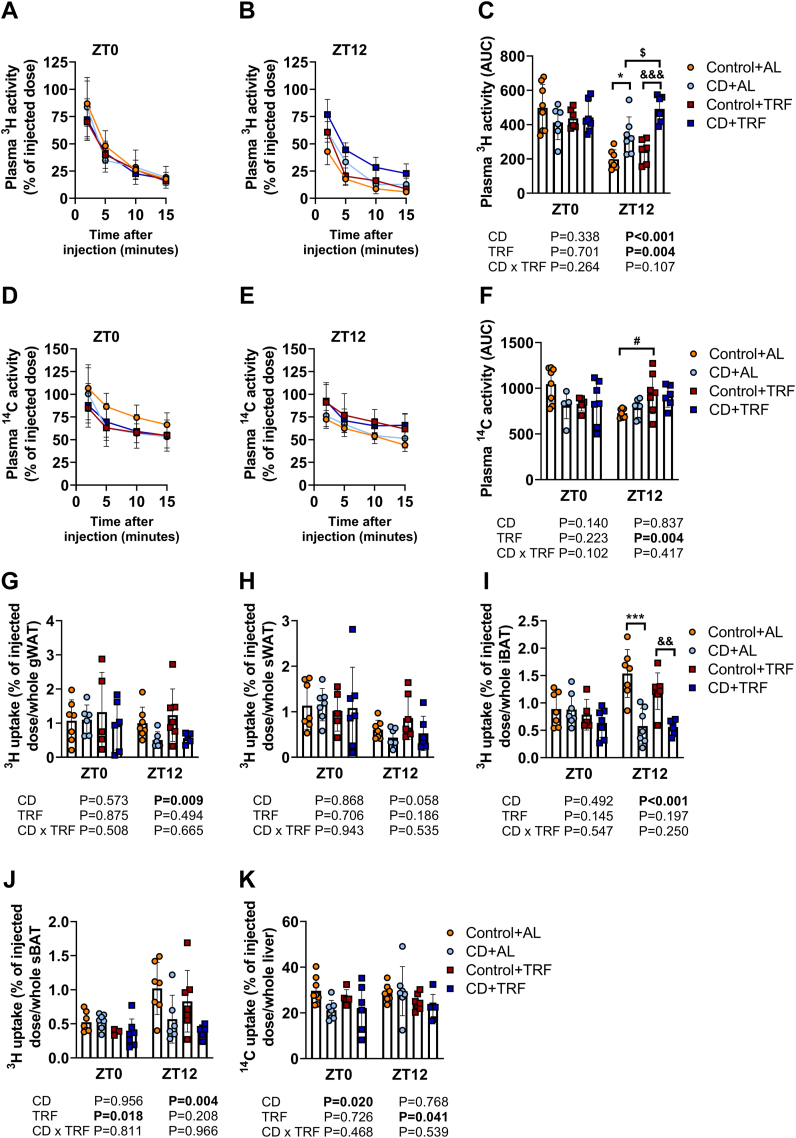
**Diurnal variation in clearance and uptake of triglyceride-rich lipoproteins (TRLs) and TRL remnants.** APOE∗3-Leiden.CETP mice were exposed to 6-h phase advancement every 3 days (circadian disturbance; CD) or regular 12:12 light–dark cycle (Control), while having either *ad libitum* food access (AL) or food access during the dark phase only (time-restricted feeding; TRF) for a total duration of 14 weeks. After 14 weeks, mice were injected with TRL-like particles double-labelled with glycerol tri [^3^H]oleate and [^14^C]cholesteryl oleate at ZT0 and 12 to assess plasma decay of (**A, B, C**) [^3^H]oleate (n = 5–8 mice/group/time point) and (**D, E, F**) [^14^C]cholesteryl oleate and [^3^H]oleate uptake by (**G**) gonadal white adipose tissue (gWAT), (**H**) subcutaneous WAT (sWAT), (**I**) interscapular brown adipose tissue (iBAT), (**J**) and subscapular BAT (sBAT), and (**K**) [^14^C]cholesteryl oleate uptake by the liver (n = 5–7 mice/group/time point). Data are presented as means ± SD. ^$^CD + AL vs. CD + TRF; ∗ Control + AL vs. CD + AL; ^&^Control + TRF vs. CD + TRF. ^$,^∗^,#^P < 0.05; ^&&^ P < 0.01; ∗∗∗^,&&&^ P < 0.001, according to two-way ANOVA and following Tukey's multiple-comparison test.

Next, we investigated if altered lipid handling by peripheral organs could explain the elevated plasma TG levels and postprandial TG excursions at ZT12 caused by CD and/or the reduced plasma TC levels upon TRF during CD. We therefore assessed plasma clearance and tissue uptake of glycerol tri [^3^H]oleate and [^14^C]cholesteryl oleate from intravenously-injected TG-rich lipoprotein-like particles at the study endpoint at ZT0 and ZT12 (24 and 12 h after the last phase shift for ZT0 and ZT12, respectively). In line with delayed postprandial TG excursions (Fig. 5e and f), CD delayed the plasma decay of ^3^H-derived activity at ZT12 regardless of TRF (CD effect at ZT12 by two-way ANOVA: P < 0.001) (Fig. 6a–c), an effect explained by reduced uptake of [^3^H]oleate by WAT and BAT (CD effect at ZT12 by two-way ANOVA: P = 0.009, P = 0.058, P < 0.001, and P = 0.004 for gonadal (g)WAT, subcutaneous (s)WAT, interscapular (i)BAT, and subscapular (s)BAT, respectively) (Fig. 6g–j). In contrast, plasma decay of ^14^C-derived activity was unchanged with CD (Fig. 6d–f) and hepatic uptake of [^14^C]cholesteryl oleate was reduced at ZT0 regardless of TRF (CD effect at ZT0 by two-way ANOVA: P = 0.020) (Fig. 6k), indicating that hepatic TRL remnant uptake does not explain the reduction in plasma TC levels caused by CD.

**Fig. 7:**
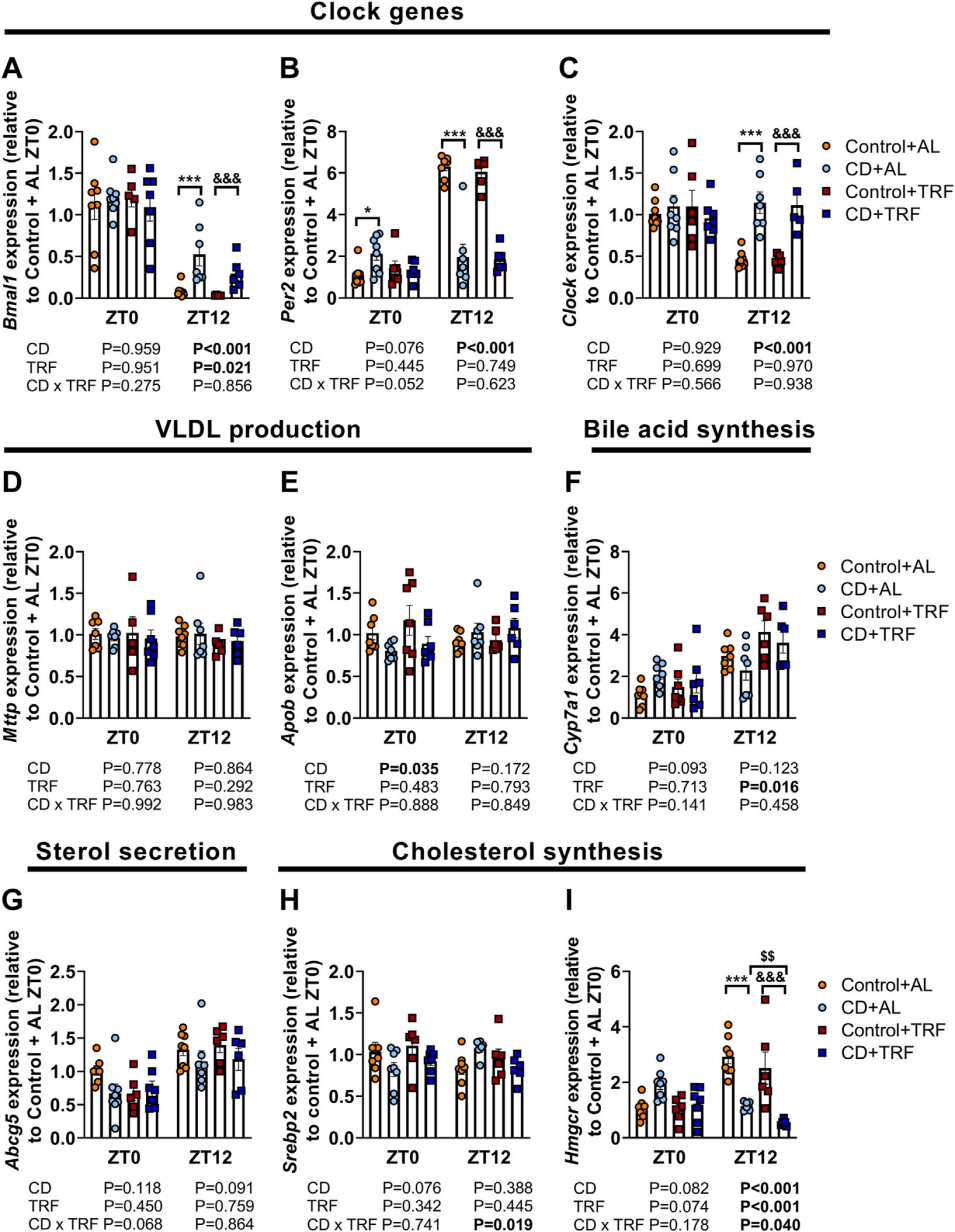
**Hepatic expression of genes involved in the core clock machinery and cholesterol metabolism.** APOE∗3-Leiden.CETP mice were exposed to 6-h phase advancement every 3 days (circadian disturbance; CD) or regular 12:12 light–dark cycle (Control), while having either *ad libitum* food access (AL) or food access during the dark phase only (time-restricted feeding; TRF) for a total duration of 14 weeks. Hepatic gene expression of (**A**) brain and muscle Arnt-like protein-1 (*Bmal1*), (**B**) period 2 (*Per2*), (**C**) circadian locomotor output cycles kaput (*Clock*), (**D**) microsomal triglyceride transfer protein (*Mttp*), (**E**) apolipoprotein B (*Apob*), (**F**) cholesterol 7 alpha-hydroxylase (*Cyp7a1*), (**G**) ATP-binding cassette sub-family G member 5 (*Abcg5*), (**H**) sterol regulatory element-binding protein 2 (*Srebp2*), and (**I**) 3-hydroxy-3-methyl-glutaryl-coenzyme A reductase (*Hmgcr*) at *Zeitgeber* time (ZT) 0 and 12, as determined by quantitative polymerase chain reaction, normalized to glyceraldehyde-3-phosphate dehydrogenase (*Gapdh*) and shown relative to the expression in control AL ZT0 (n = 5–8 mice/group/time point). Data are presented as means ± SD. ^$^CD + AL vs. CD + TRF; ∗ Control + AL vs. CD + AL; ^&^Control + TRF vs. CD + TRF. ∗P < 0.05; ^$$^ P < 0.01; ∗∗∗^,&&&^ P < 0.001, according to two-way ANOVA and following Tukey's multiple-comparison test.

Since the liver is the primary organ of cholesterol production and secretion, we next investigated hepatic expression of genes involved in cholesterol metabolism besides those involved in the cellular clock. CD profoundly altered clock gene expression at ZT12, suggestive of shifts in hepatic rhythms (Fig. 7a–c). The shift in *Per2* expression at ZT0 tended to be attenuated by TRF (CD-TRF interaction at ZT0 with two-way ANOVA: P = 0.052). Both CD and TRF did not markedly alter the expression of microsomal triglyceride transfer protein (*Mttp*) and apolipoprotein B (*Apob*), genes encoding proteins that are involved in VLDL production, cholesterol 7 alpha-hydroxylase (*Cyp7a1*) that regulates bile acid synthesis, or ATP-binding cassette sub-family G member 5 (*Abcg5*), a regulator of sterol secretion towards the bile (Fig. 7d–g). Interestingly, TRF did attenuate sterol regulatory element-binding protein 2 (*Srebp2*) expression at ZT12 (CD-TRF interaction at ZT12 by two-way ANOVA: P = 0.019) (Fig. 7h), and the CD + TRF group showed a pronounced reduction in *Hmgcr* expression at both ZT0 and ZT12 compared with the CD + AL group, albeit non-significantly at ZT0, indicating reduced cholesterol synthesis (Fig. 7i).

## Discussion

**Fig. 8:**
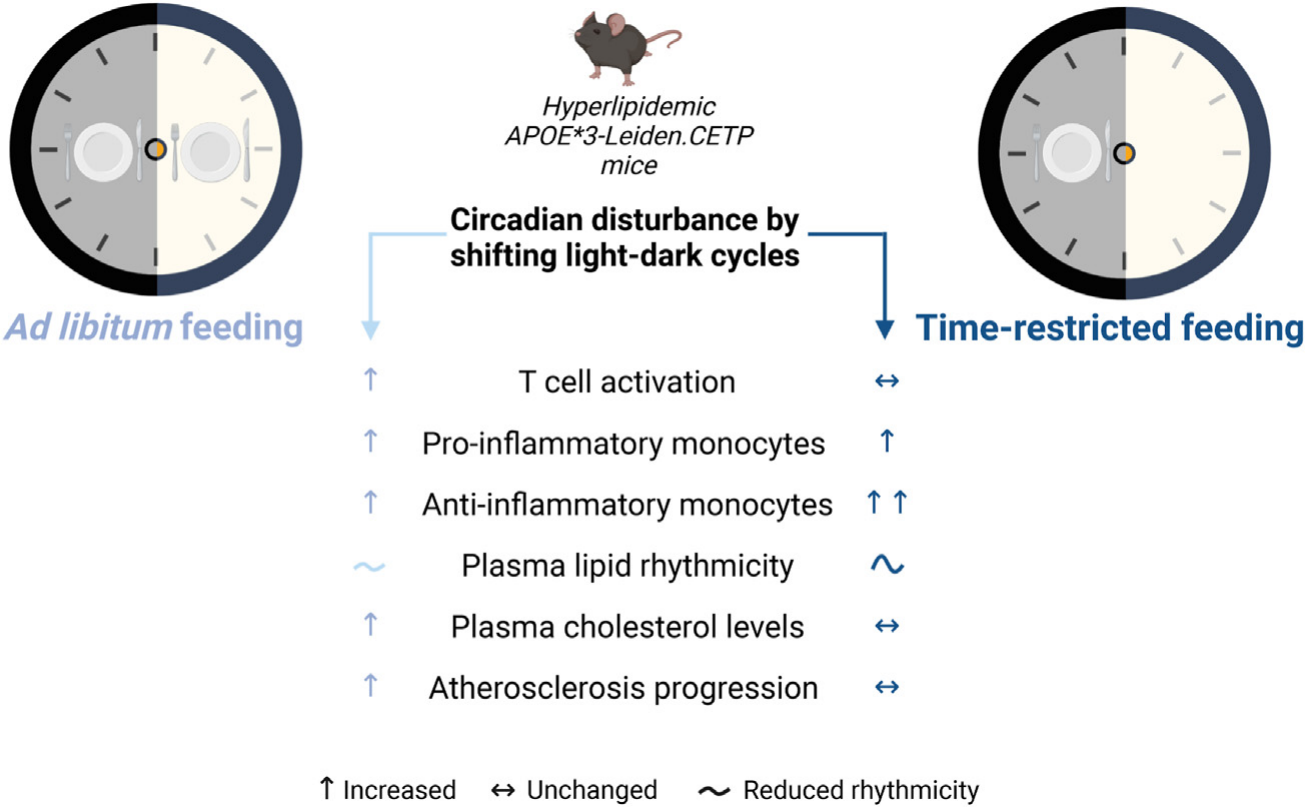
**Summarizing overview.** Created with BioRender.com.

CD, which frequently occurs during shift work, has been associated with increased asCVD risk in humans, but evidence for the effectiveness of prevention strategies is lacking. Here, we assessed if TRF is a viable strategy to prevent elevated asCVD risk caused by CD. To this end, CD was modelled in APOE∗3-Leiden.CETP mice through repeated shifts in the light–dark cycles, which resulted in a non-significant elevation in atherosclerotic lesion size and significantly increased macrophage content within the lesions when mice were fed AL. We demonstrate that restricting food intake to the dark phase completely prevents the increase in atherosclerotic lesion area and macrophage content caused by CD. A summarizing overview is displayed in Fig. 8.

Inflammatory processes involved in atherogenesis are under control of the circadian clock, including the abundance of circulating immune cells in both mice and humans.^9^^,^^30^ Disturbance of circadian rhythms by genetic deletion of clock genes in mice results in a pro-inflammatory immune cell profile as well as higher macrophage infiltration in atherosclerotic lesions.^31^, ^32^, ^33^ In our study, we observed that CD alters the immune system by inducing shifts in monocyte profile, including a time-dependent increase in pro-inflammatory classical monocytes and anti-inflammatory non-classical monocytes, as well as depleting eosinophil levels, and causing an activated T cell profile in blood. While these findings might indicate migration of immune cells towards atherosclerotic lesions to promote atherogenesis, future studies should assess the local effects of monocytes, eosinophils, and T cells to atherosclerosis lesion development in the context of CD. It is interesting to note that CD induced both pro-inflammatory classical monocytes and anti-inflammatory non-classical monocytes, albeit at different time points. The net effect on atherogenesis is, however, difficult to predict, as it also highly depends on (rhythms in) recruitment of monocytes to the vessel wall. Opposed to AL feeding, TRF caused a continuous increase in the number non-classical monocytes and appeared to prevent the activation of T cells in blood upon CD. In addition, we observed that CD elevated (postprandial) TG levels, coinciding with reduced lipid uptake by WAT and BAT at the onset of the dark phase. Accordingly, plasma lipid levels and lipid uptake by BAT follow a pronounced diurnal rhythm,^29^^,^^34^ and CD was previously shown to attenuate rhythmic lipid uptake by BAT and elevate plasma lipid levels.^35^^,^^36^ This suggests that restoring diurnal rhythms in adipose tissue is a potential therapeutic strategy to prevent elevations in plasma TGs caused by CD. These observations in mice are very comparable to what has previously been reported for humans, as levels of circulating immune cells and lipids also follow a diurnal rhythm, and shift work has been associated with systemic inflammation and dyslipidaemia.^29^^,^^37^, ^38^, ^39^, ^40^, ^41^, ^42^

In our study, TRF did not prevent all of the negative effects caused by CD, such as the increase in pro-inflammatory monocytes or the elevated (postprandial) plasma TG levels. Of all tissues, the liver appears to be most responsive to altered feeding patterns.^43^ In line with this notion, TRF lowered markers of hepatic cholesterol synthesis during CD, which possibly underlies the attenuation of hypercholesterolaemia. Avoiding food intake during the inactive phase by applying TRF may thereby prevent disturbances in hepatic lipid metabolism during CD.

Notably, TRF by itself did not attenuate asCVD risk factors, such as hyperlipidaemia, nor did it prevent atherosclerosis in our study. In fact, TRF by itself delayed plasma clearance of TRL remnants, albeit without altering plasma TC levels. This may indicate that TRF is only beneficial when diurnal eating patterns are disturbed by for example during CD or upon diet-induced obesity.^44^^,^^45^ In humans, TRE has shown to improve cardiometabolic health, but the question remains to what extent the effects of TRE surpass those of calorie reduction.^46^ We now show that independent of total food intake, TRF attenuates atherosclerosis development in the context of CD. We therefore propose that TRE could be a potential strategy for the primary prevention of elevated asCVD risk in shift workers, which warrants future study in humans. In this respect, it is reassuring that a recent pioneering study demonstrated that TRE is safe and feasible in a population of shift workers.^47^ Future studies should assess the extent to which the protective effects of TRE are dependent on the duration of the eating window and/or the time of day that the eating window is applied to. Possibly this needs to be sorted out on an individual basis for human shift workers.

A limitation of mice as model for atherosclerosis development is that atherosclerotic lesions usually manifest in the aorta and proximal great vessels, and rarely in the coronary vessels. In addition, lesions do not seem to rupture nor cause thrombus formation or haemorrhage in APOE∗3-Leiden.CETP mice.^48^ Therefore, APOE∗3-Leiden.CETP mice cannot be used for studying cardiovascular events such as heart failure. Interestingly, TRF was previously shown to improve cardiac function through modulation of the circadian clock and attenuated CD in Drosophila.^49^ It would be of much interest to study what the effects of CD on cardiac function are and whether TRF has a protective effect. Furthermore, female APOE∗3-Leiden.CETP mice are not prone to develop obesity and related pathologies, such as insulin resistance and intramuscular lipid accumulation. Future studies could assess the combination of CD and TRF in a mouse model prone to spontaneous obesity development.

In addition to timed food intake, other *Zeitgebers* could be exploited to mitigate elevated asCVD risk during shift work. For example, physical activity acts as a *Zeitgeber*, as timed exercise can shift behavioural rhythms in mice by modulating the molecular muscle clock.^50^^,^^51^ Timed exercise alone or in combination with TRE might therefore be a promising strategy to minimize adverse effects of CD on cardiometabolic health. Optimization of timed exercise and eating interventions likely requires personalized adjustments, as high inter-individual variability exists in the circadian system and shift work tolerance, i.e., the ability to adapt to shift work without adverse consequences such as digestive troubles, persisting fatigue, and sleep alteration. This variation in circadian rhythms and shift work tolerance is caused by factors such as genetics, age, sex, and chronotype,^52^ as well as the time window at which the night shift takes place, whether the shifts are permanent or rotating, the total number of consecutive shifts, and the work place conditions including lighting conditions.^53^ Similarly, responsiveness to circadian interventions may be variable as one study demonstrated that circadian phase shifts caused by timed exercise vary with chronotype.^54^ Future research should therefore characterize inter-individual differences in the phase-shifting response to timed eating and exercise, which could contribute to development of personalized interventions for primary disease prevention in shift working populations.

## Contributors

W.I.H.P., M.S., P.C.N.R., and S.K. conceived and designed the research; W.I.H.P., M.S., M.M., H.E.T., R.A.L., A.C.M.P., T.C.M.S., M.A.C.D., I.B., W.G.V., L.A.B., B.W.v.O., and S.K. performed the research and acquired the data, W.I.H.P., H.E.T., B.W.v.O., and S.K. analyzed the data, W.I.H.P. and S.K. verified the data, W.I.H.P., M.S., P.C.N.R., and S.K. interpreted the data, and L.W.M.K., M.E.T.D., E.L., P.C.N.R., and S.K. acquired funding. All authors were involved in the drafting and revising of the manuscript, and all authors read and approved the final version of the manuscript.

## Data sharing statement

The data that support the findings of this study are available from the corresponding author upon request.

## Declaration of interests

The authors declare that the research was conducted in the absence of any commercial or financial relationships that could be construed as a potential conflict of interest.
