# Supplementary material for: Time-restricted feeding attenuates hypercholesterolaemia and atherosclerosis development during circadian disturbance in APOE∗3-Leiden.CETP mice
Source: eBioMedicine. 2023 Jun 23;93:104680. doi: 10.1016/j.ebiom.2023.104680 (PMC10320519; doi:10.1016/j.ebiom.2023.104680)
Supplement: Figs. S1–S3 and Tables S1–S2 [file mmc1.docx]

**Time-restricted feeding attenuates hypercholesterolaemia and atherosclerosis development during circadian disturbance in APOE*3-Leiden.CETP mice**

Wietse In het Panhuis^1,2^, Milena Schönke^1,2^, Melanie Modder^1,2^, Hannah E. Tom^1,2^, Reshma A. Lalai^1,2^, Amanda C.M. Pronk^1,2^, Trea C.M. Streefland^1,2^, Linda W.M. van Kerkhof^3^, Martijn E.T. Dollé^3^, Marie A.C. Depuydt^4^, Ilze Bot^4^, Winnie G. Vos^5,6,7^, Laura A. Bosmans^5,6,7^, Bram W. van Os^5,6,7^, Esther Lutgens^5,6,7,8^, Patrick C.N. Rensen^1,2^, and Sander Kooijman^1,2^*

^1^Division of Endocrinology, Department of Medicine, Leiden University Medical Center, Leiden, The Netherlands

^2^Einthoven Laboratory for Experimental Vascular Medicine, Leiden University Medical Center, Leiden, The Netherlands.

^3^Centre for Health Protection, National Institute for Public Health and the Environment, Bilthoven, The Netherlands

^4^Leiden Academic Centre for Drug Research, Division of Biotherapeutics, Leiden University, Leiden, The Netherlands

^5^Amsterdam UMC, location AMC, University of Amsterdam, Department of Medical Biochemistry, Amsterdam, The Netherlands

^6^Amsterdam Cardiovascular Sciences, Atherosclerosis & Ischemic Syndromes, Amsterdam, The Netherlands

^7^Amsterdam Immunity and Infection, Amsterdam, The Netherlands

^8^Department of Cardiovascular Medicine, Mayo Clinic, Rochester, Minnesota, USA

*Corresponding author: Sander Kooijman
Visiting address: Albinusdreef 2, 2333ZA Leiden, The Netherlands
Posting address: P.O. Box 9600, 2300 RC Leiden, The Netherlands
E-mail: [s.kooijman@lumc.nl](mailto:s.kooijman@lumc.nl) / Phone: +31 (0)71 52 63989

**Table of contents**

Table S1. Three-way ANOVA of diurnal triglyceride levels……………………………………………………..3

Table S2. Three-way ANOVA of diurnal total cholesterol levels………………………………………….……..3

Figure S1. Methodological description atherosclerosis quantification……………………………………………4

Figure S2. Voluntary locomotor activity and sleep………………………………………………………………..5

Figure S3. Aortic expression of genes involved in inflammation, leukocyte recruitment, and oxidative stress……………………………………………………………………………………………………………….6

**Table S1. Atherosclerotic lesion severity scoring, according to the guidelines of the American Heart Association adapted for mice.**

| **Lesion Type** | **Characteristics** |
| --- | --- |
| I (mild) | Early fatty streak: up to and including 10 foam cells in the intima |
| II (mild) | Regular fatty streak: over 10 foam cells present in the intima |
| III (mild) | Mild plaque: extension of foam cells into the media and covered by a fibrotic cap |
| IV (severe) | Moderate plaque: a more progressive lesion; fibrosis of the media, without loss of architecture |
| V (severe) | Severe plaque: The media is severely damaged, elastic lamina are broken. Often visible: cholesterol clefts/crystals, mineralisation (calcium) and necrosis |

**Table S2. One-way ANOVA of diurnal triglyceride levels.** CD=circadian disturbance, TRF=time-restricted feeding

| **Group** | **F** | **P-value (time effect)** | **R squared** |
| --- | --- | --- | --- |
| Control+AL | 16.43 | <0.001 | 0.474 |
| CD+AL | 2.009 | 0.103 | 0.103 |
| Control+TRF | 31.38 | <0.001 | 0.649 |
| CD+TRF | 10.13 | <0.001 | 0.370 |

**Table S3. One-way ANOVA of diurnal total cholesterol levels.** CD=circadian disturbance, TRF=time-restricted feeding

| **Group** | **F** | **P-value (time effect)** | **R squared** |
| --- | --- | --- | --- |
| Control+AL | 1.893 | 0.121 | 0.094 |
| CD+AL | 0.064 | 0.992 | 0.004 |
| Control+TRF | 1.696 | 0.161 | 0.091 |
| CD+TRF | 2.66 | 0.040 | 0.134 |

**
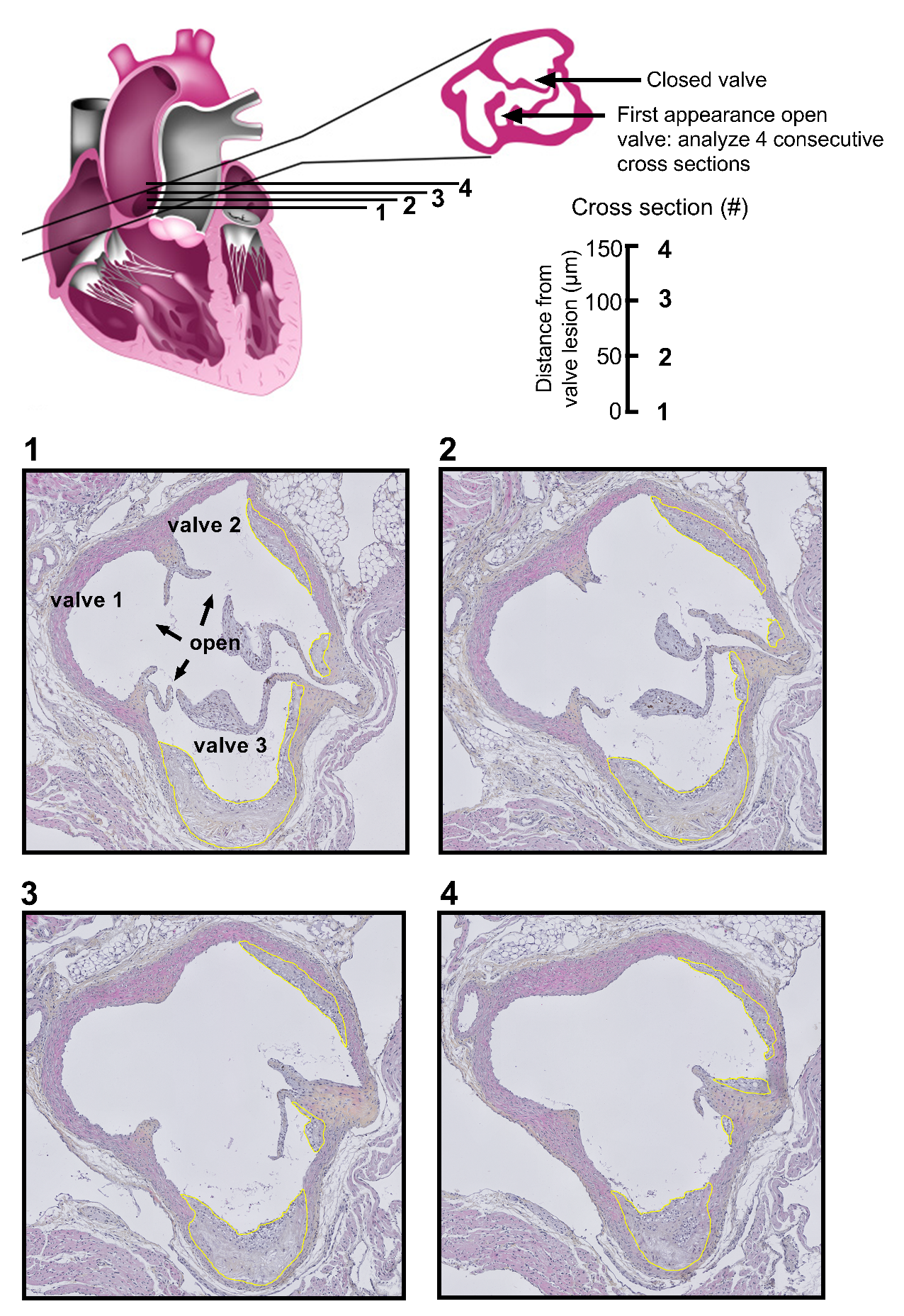
**

**Figure S1. Methodological description atherosclerosis quantification.** Cross sections (5 µm thickness) were made perpendicular of the aortic root and stained for haematoxylin-phloxine-saffron. For each valve, four consecutive cross sections (50 µm distance between cross sections) were analysed, starting at the first appearance of that valve. In the figure above, all valves appeared open on cross section 1. All lesions within each valve were analysed for area by drawing a region of interest (yellow drawings) and for type and lesion severity by subjective scoring (mild; type I-III and severe; type IV-V). Mean area and severity was calculated per mouse.


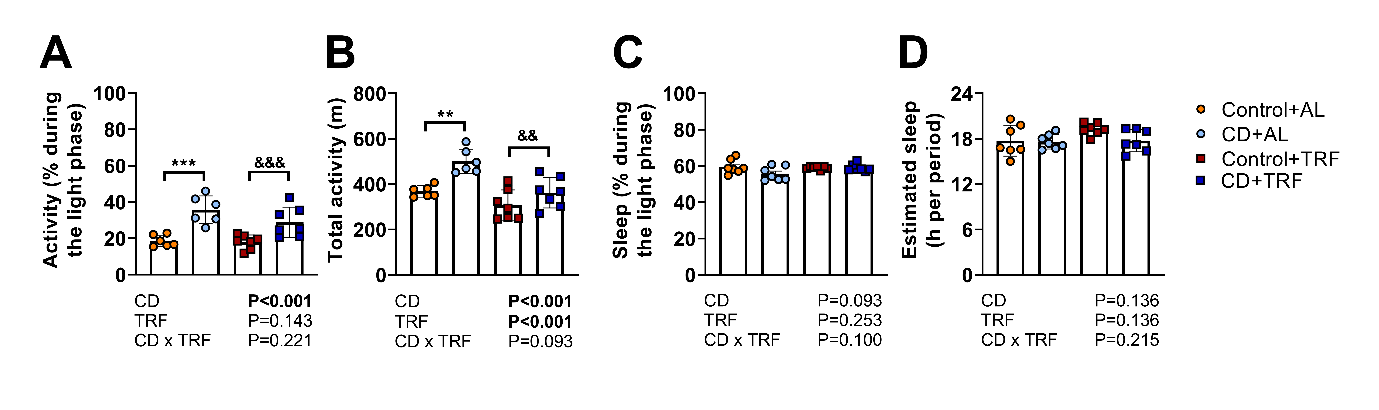


**Figure S2. Voluntary locomotor activity and sleep.** APOE*3-Leiden.CETP mice were exposed to 6-hour phase advancement every 3 days (circadian disturbance; CD) or regular 12:12 light-dark cycle (Control), while having either *ad libitum* food access (AL) or food access during the dark phase only (time-restricted feeding; TRF) for a total duration of 14 weeks. During the sixth cycle of phase shifts, mice were housed in metabolic home-cages for continuous measurement of (**A, B**) voluntary locomotor activity and (**C, D**) estimated sleep, after which the percentage of each parameter during the light phase and the total levels were calculated (n=4-7 cages of 2-3 mice/group). Data are presented as means ± SD. * Control + AL vs. CD + AL; ^&^Control + TRF vs. CD + TRF. **^,&&^ p<0.01; ***^,&&&^ p<0.001, according to two-way ANOVA and following Tukey’s multiple-comparison test.


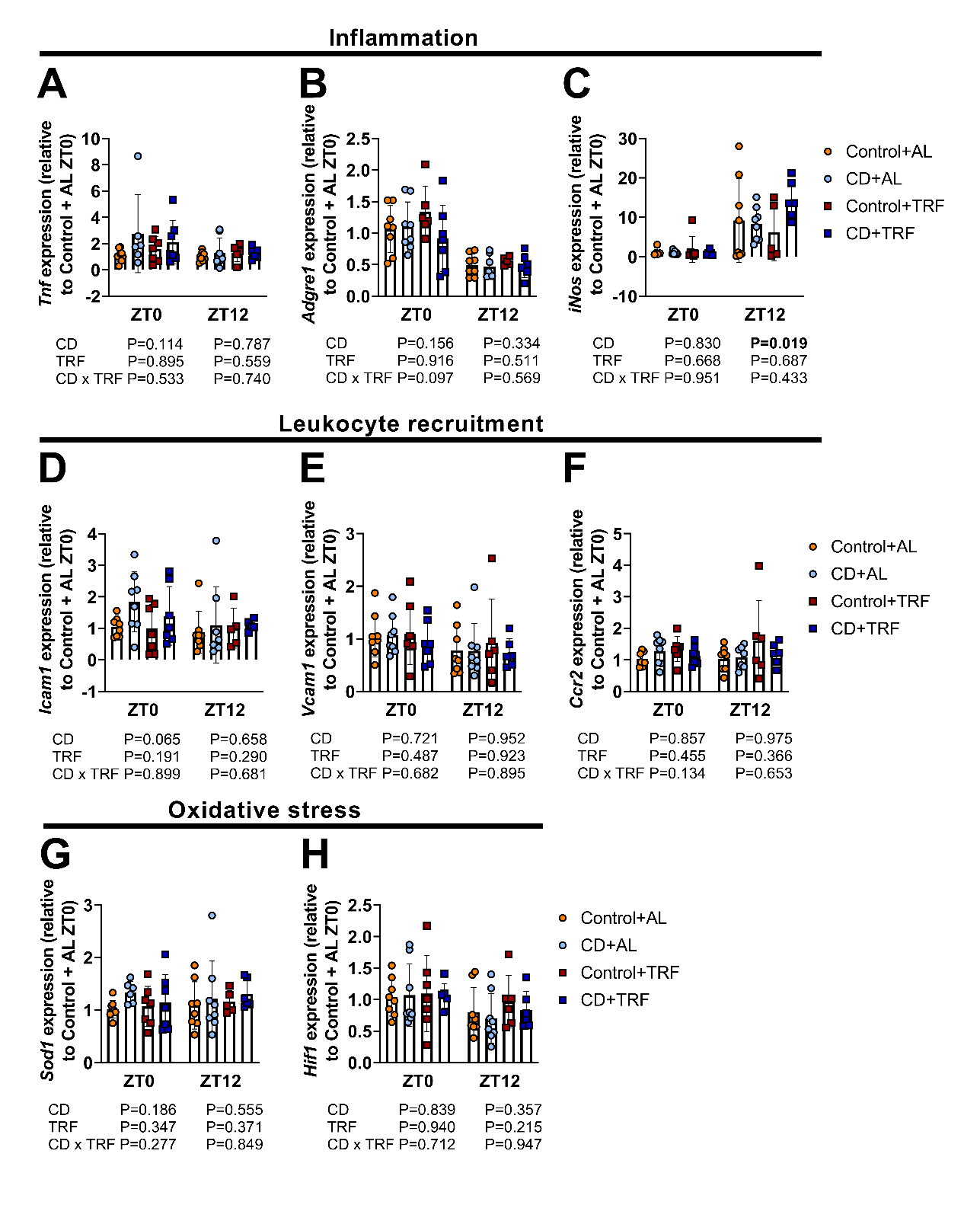


**Figure S3. Aortic expression of genes involved in inflammation, leukocyte recruitment, and oxidative stress.** APOE*3-Leiden.CETP mice were exposed to 6-hour phase advancement every 3 days (circadian disturbance; CD) or regular 12:12 light-dark cycle (Control), while having either *ad libitum* food access (AL) or food access during the dark phase only (time-restricted feeding; TRF) for a total duration of 14 weeks. Aortic gene expression of (**A**) tumor necrosis factor alpha (*Tnf*), (**B**) adhesion G protein-coupled receptor E1 (*Adgre1*), (**C**) inducible nitric oxide synthase (*iNos*), (**D**) intercellular adhesion molecule 1 (*Icam1*), (**E**) vascular cell adhesion molecule 1 (*Vcam1*), (**F**) C-C chemokine receptor type 2 (*Ccr2*)*,* (**G**) superoxide dismutase 1 (*Sod1*), and (**H**) hypoxia inducible factor 1 subunit alpha (*Hif1*) at *Zeitgeber* time (ZT) 0 and 12, as determined by quantitative polymerase chain reaction, normalized to β2 microglobulin (*B2m*) and shown relative to the expression in control AL ZT0 (n=5-8 mice/group/time point). Data are presented as means ± SD. According to two-way ANOVA and following Tukey’s multiple-comparison test.
